# The Effects of Empowerment Education on Daily Dairy Intake in Community-Dwelling of Older Asian Women

**DOI:** 10.3390/ijerph18094659

**Published:** 2021-04-27

**Authors:** Pei-Ti Hsu, Jeu-Jung Chen, Ya-Fang Ho

**Affiliations:** 1 Department of Nursing, Ching Kuo Institute of Management and Health, Keelung 20301, Taiwan; bettyhsu1@gmail.com; 2 Department of Rehabilitation, Taiwan Adventist Hospital, Taipei 10556, Taiwan; lauraapplepie@gmail.com; 3 School of Nursing, China Medical University, Taichung 406040, Taiwan

**Keywords:** older women, empowerment, dairy intake behavior, community, Asian

## Abstract

A scarcity in the intake of dairy products in older women begets a wide range of musculoskeletal problems, especially osteoporosis. However, dairy products are often not consumed in sufficient quantities in Eastern societies. This study used empowerment education to improve daily dairy intake in 68 older Asian women in the community through a quasi-experimental study design. The 34 participants in the experimental group took part in empowerment education programs that included lectures, sharing sessions, situation dramas, and cooking activities, for two hours per week for 6 weeks. The 34 participants in the control group had no interventions. The generalized estimating equation was used to evaluate the intervention’s effectiveness. The test was conducted for the two groups at 1 and 12 weeks after the completion of the lessons. We used daily dairy intake self-efficacy, intention, and behavior scale to measure the outcome. The change in the experimental group’s daily dairy intake self-efficacy and intention score at post-1 week and post-12 weeks was higher compared to the control group, but the dairy intake behavior was only changed at post-12 weeks. The empowerment education was effective in encouraging older women to change their dairy intake behavior and improved their dairy intake self-efficacy and intentions.

## 1. Introduction

Calcium is required for a number of specific roles in the body as all cells require calcium to remain viable [1]. Calcium may be related to the cause of many diseases, with evidence suggesting that calcium supplementation can reduce blood pressure and has lipid-lowering effects, thereby reducing the risk of cardiovascular disease [1,2]. In addition, another study found low dietary intake of calcium results in severe periodontal disease [3]. One cause or important contributing factor in osteoporosis is a dietary deficiency of calcium [4]. Among adult people, low dietary calcium intakes are thought to produce osteoporosis through secondary hyperparathyroidism and increased bone turnover [5], leading to increased susceptibility to fractures [6]. The fracture may lead to disability in older adults which, in turn, can cause a decline in quality of life [7,8,9]. Moreover, it also represents a substantial cause of mortality and morbidity, particularly for postmenopausal women [10,11]. Due to several physiological factors, postmenopausal older women are prone to develop osteoporosis [1,12,13]. Among adults aged 65 years and over in Taiwan, the prevalence of hip fracture is 5.2% for women, well above the 2.4% for men, furthermore, as high as 8.5% of women aged 75 years and over had suffered hip fractures, which was also higher than the 3.5% in men of the same age [14]. Hip fracture is the costliest result as it always requires hospitalization, is fatal in 20% of cases, and permanently disables a further 50% [6].

It is known that nutrition, especially calcium supplementation, has an important effect on maintaining bone health [15]. Dairy products are an important source of calcium [1,16,17]. Dairy calcium is highly bioavailable and accounts for more than 50% of total calcium intake [18]. Consumption of calcium-rich dairy products has been shown to reduce the loss of bone [18,19]. Studies have pointed out the existence of a significant positive association between dairy product consumption and bone properties in older adults [20]. However, the results of the 2013–2016 Nutrition and Health Survey in Taiwan indicate that among older women aged 65 years and over, as much as 87% of this demographic consume less than one serving of dairy products daily [21]. The inadequate consumption of dairy products is a significant reason for insufficient calcium intake in older women. One study revealed that each additional serving of milk per day in postmenopausal women was associated with a significant 8% lower risk of hip fractures [22]. Therefore, these women should be encouraged to consume more dairy products. However, due to differences between Western and Eastern cultures, dairy products are not consumed in sufficient quantities in many Eastern societies. Dairy products, despite being excellent sources of calcium, may not be often consumed within traditional Asian diets because of lack of familiarity [17]. Their reasons tend to be due to unawareness of the importance of consuming dairy products for bone health rather than dislike of ingesting them [23]. If older women can understand that dairy products are an important source of calcium, and that it is fairly easy to accommodate these products in one’s diet, it is likely that they would make behavioral changes with respect to their daily dairy intake behavior.

Empowerment is a dynamic process, in which power is transferred from the empowerer to the empowered; through listening, dialogue, reflection, and shared participation, and by establishing trust and partnerships and facilitating the exchange of information, the empowered gains the opportunities and right to make their own decisions [24]. The improvement of one’s abilities is another important concept of empowerment; an individual participating in the empowerment process must possess sufficient capability and be able to make his or her own choices and decisions, and as such, empowerment is a process in which an individual gains power and capability [25,26]. Chen et al. (2011) conducted a systematic literature review of 18 relevant studies and indicated that the empowerment principle should encompass awareness awakening, open communication, the provision of the necessary information, and mutual participation [27]. Awareness awakening refers to the process in which an individual must first become aware of his or her intrinsic motivations, sense his or her needs and problems, and develop the intention to change. In this process, mutual participation is seen as a crucial dimension, the empowerer adopts open communication with the empowered; the empowerer can provide the necessary information and create a supportive environment. The empowerment concept has been widely applied in self-care for a wide variety of diseases, and the results have been positive [28,29,30]. At present, however, few studies have evaluated the implementation and effectiveness of health education aimed at influencing the daily dairy intake behavior of older Asian women. To this end, our study adopted the empowerment concept to develop an educational strategy for influencing the daily dairy intake behavior of older women, and explored its effects on self-efficacy, intention, and behavior relating to daily dairy intake among older women.

## 2. Materials and Methods

### 2.1. Study Design and Participants

A quasi-experimental study was conducted in Keelung City’s Renai Distric, Taiwan. G*Power software (Heinrich Heine University. Dusseldorf, North Rhine-Westphalia, Germany) was used to estimate the required sample size. According to the results of previous studies [28], we estimated that an appropriate sample size for the analysis was 62 participants, based on alpha level = 0.05, medium effect size = 0.3, and power = 0.8 in repeated-measures ANOVA. Considering a sample loss rate of 10%, we recruited 68 older women, and participants were randomly assigned to either experimental or control groups by drawing lots, each group being assigned 34 participants. The study was approved by the Taipei Hospital Institutional Review Board (IRB NO. TH-IRB-0017-0026). Participants were eligible to participate in the study if they were 65 years old and over; had the ability to walk unassisted with no mobility issues; had a Short Portable Mental Status Questionnaire (SPMSQ) score of 8 points or higher. The Barthel scale was used to assess the participants’ daily living activities, and only those with a score of 91 points or higher were included in the study. No participants withdrew from the study, all the participants completed the pretest (T0), post-1 week (T1), and post-12 weeks (T2).

### 2.2. Empowerment Program

The education program developed in this study was designed based on the empowerment principles proposed by Chen et al. (2011) [27]. Our study proposed the following four strategies. Dialogue and reflection: researchers will converse with, listen to, and care for participants and their feelings. In dialogue, a bilateral exchange of experiences and knowledge between participants and researchers takes place, creating a partnership based on mutual trust and respect in which open communication is used to help participants to perform reflections and awaken their intrinsic motivations. Sharing of life experiences: this strategy allows participants to share their dietary lifestyles and experiences and establish support networks and resources, creating a supportive environment. Provision of information and counseling: researchers provide information on dairy intake, enhance participants’ knowledge of related topics, and continue to care for the participants who have completed their courses through consultations, during which they can address questions and help the participants to overcome problems relating to milk consumption. The focus was on the co-creation of knowledge rather than just a transference of knowledge. Group motivation: this strategy allows participants to encourage and support each other, with group discussions being implemented to enable the acquisition of problem-solving skills.

### 2.3. Procedures

**Figure 1:**
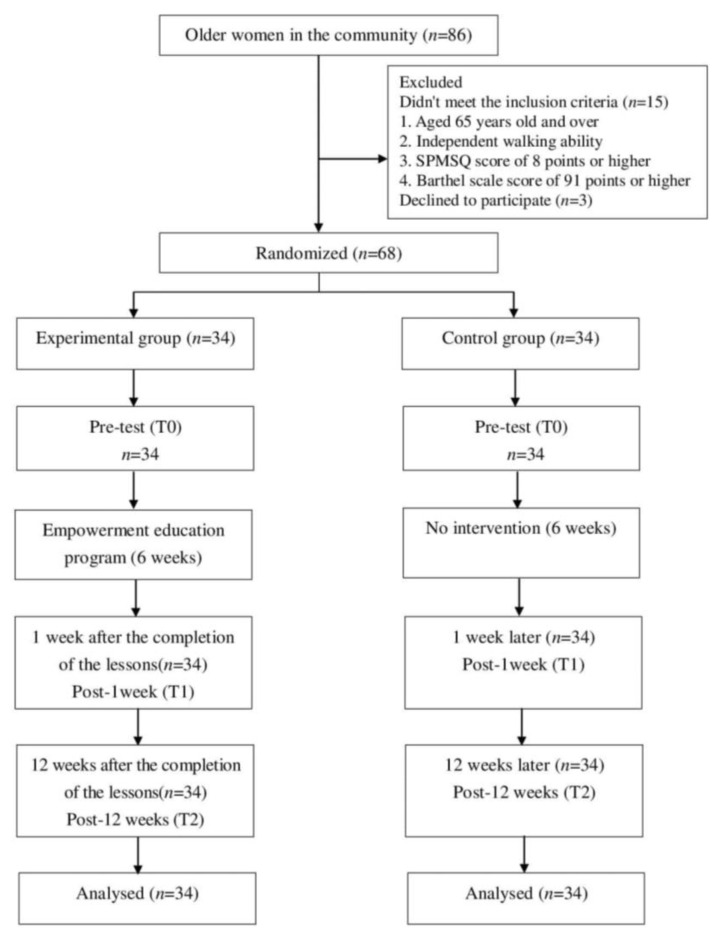
Flow diagram.

The empowerment education program comprised six lessons, conducted once a week, for two hours each time, for a total of six weeks. Presented in order, the themes are “Keeping osteoporosis away,” “Drink more milk to stay healthy,” “Cheering for your friends, and yourself,” “I’m a detective,” “My dairy products, my decision,” and “Carrying on with my efforts.” The education program and telephone contact were carried out by the first author, who is an expert in nursing education for older adults. Each lesson started with an introduction, during which the first author would converse with the participants, listen to their feelings, and provide encouragement and support; they would then cover the theme of the week, and conduct group discussion and sharing sessions after completing the lesson. The content of the empowerment education program is shown in Table 1 (see supplementary file for more detailed content Table S1). In this period, each older woman in the experimental group was given telephone contact once a week, for a total of 6 times. In the telephone contact, the older women’s problems were listened to and answered. The control was not subjected to any interventions. The researchers first conducted a pretest (T0) before the intervention. The post-1 week (T1) and post-12 weeks (T2) were conducted for the two groups at 1 and 12 weeks after the completion of the lessons (Figure 1).

### 2.4. Instruments

#### 2.4.1. Personal Characteristics

Personal characteristics of baseline include age, marital status, education level, residential status, financial condition, and self-perceived health. The study found that social support had a significant contributing role to daily dairy product consumption, and dairy products were significantly related to nutritional status [31,32]. Therefore, we included social support and nutritional status into the baseline. Social support was evaluated using the Chinese version of the Emotional Social Support scale (ESS), which primarily evaluates the degree to which research participants feel that they have benefited from emotional social support. This scale contains six items that are scored on a scale of 1 to 4, with the lowest and highest possible total scores being 6 and 24, respectively. A higher score indicates that the participant has received a higher degree of social support. The scale has a Cronbach’s α value of 0.82, indicating good reliability. We also used the Mini Nutritional Assessment (MNA) scale Taiwan Version-2 to assess the nutritional status of the participants. This scale was specifically designed for the older patient; the scale contains 18 items, with the highest possible total score being 30. The final score allows grading the nutritional status: scores above 24, good status; scores 23.5–17, risk of malnutrition; scores below 17, malnutrition [33].

#### 2.4.2. Outcome Measurement

We used daily dairy intake self-efficacy, intention, and behavior scales to measure the outcome. These three scales have been reviewed, pretested, and revised by experts for validity before being used in the study. The validity assessment was primarily performed by six invited experts from the fields of nutrition, public health, and nursing.

##### Daily Dairy Intake Self-Efficacy

Three items were used to evaluate the research participants’ dairy intake self-efficacy. These items were scored on a five-point Likert scale. The scale contents are scored on a scale of 1 to 5, with the lowest and highest possible total scores being 3 and 15, respectively. A higher total score indicates better dairy intake self-efficacy. For this study, the scale had a CVI of 0.82 and Cronbach’s α of 0.92, indicating good reliability and validity.

##### Daily Dairy Intake Intention

Three items were used to evaluate the research participants’ dairy intake intention. These items were scored on a five-point Likert scale, with the lowest and highest possible total scores being 3 and 15, respectively. A higher total score indicates better dairy intake intention. For this study, the scale had a CVI of 0.86 and Cronbach’s α of 0.90, indicating good reliability and validity.

##### Daily Dairy Intake Behavior

We used one item to evaluate the dairy intake behavior of research participants. The research participants were asked to indicate the number of days, within the most recent month, that they have managed to consume one cup (240 mL) or more servings of dairy products, including cheese (1 slice about 28 g), milk, goat milk, long-life milk, calcium-fortified soy milk, yogurt, and milk powder-based milk. For this study, the scale had a CVI of 0.88.

#### 2.5. Data Analysis

The collected questionnaire data were analyzed using the SPSS for Window 20.0 statistical software (IBM Corp. Armonk, NY, USA). The distribution of the categorical variables was expressed in terms of frequency and percentage, while the distribution of the continuous variables was expressed using mean and standard deviation values. A chi-squared test and t-test were performed to determine whether the experimental and control groups’ categorical and continuous variables were homogenous at the pretest phase. Due to time considerations, a generalized estimating equation (GEE) was used to evaluate the intervention’s effectiveness on the participants’ daily dairy intake self-efficacy, intention, and behavior at pretest (T0), post-1 week (T1), and post-12 weeks (T2).

## 3. Results

### 3.1. Comparison of Experimental and Control Groups’ Personal Characteristics and Outcome Variables

A sample of 68 older women was recruited. The experimental group had an average age of 74.29 years (±9.83), and the control group had an average age of 76.88 years (±2.08), most of them were married and had an elementary level education. A chi-squared test and an independent samples t-test were performed, revealing no statistically significant differences between the two groups with respect to ESS, MNA, marital status, education level, residential status, financial condition, and self-perceived health status (*p* > 0.05). As for the outcome variables in the pretest (T0), the independent samples t-test revealed no statistically significant differences between the two groups with respect to dairy intake self-efficacy, intention, and behavior (*p* > 0.05), indicating a high degree of homogeneity (Table 2).

### 3.2. Difference in Outcome Variables between T0 and T1, T0 and T2 within the Experimental and Control Groups

The GEE was used to evaluate time effects in the two groups’ outcome variables (Table 3). The results indicate that the experimental group’s daily dairy intake self-efficacy (*p* < 0.001), intention (T0-T1: *p* = 0.004, T0-T2: *p* = 0.002), and behavior (T0-T1: *p* = 0.021, T0-T2: *p* < 0.001) score at post-1 week (T1) and post-12 weeks (T2) was, respectively, higher compared to the pretest phase (T0), with the score difference being statistically significant in both cases. The score difference of the control group did not reach statistical significance (*p* > 0.05). These findings show that, over time, empowerment education had a significant positive effect on the daily dairy intake self-efficacy, intention, and behavior of older women.

### 3.3. Difference in the Changes in Outcome Variables between the Experimental and Control Groups

GEE analyses of intergroup differences in outcome variables are displayed in Table 4. With regard to the interactions between group and time, the change in the experimental group’s daily dairy intake self-efficacy and intention score at post-1 week (T1) and post-12 weeks (T2) was higher compared to the control group, with the score difference being statistically significant, respectively (*p* < 0.001). These findings show that, over time, empowerment education had significant and continuous effects on the daily dairy intake self-efficacy and intentions of older women. With regard to dairy intake behavior, the change in the experimental group’s dairy intake behavior score at post-1 week (T1) was higher compared to the control group; however, this difference was not statistically significant (*p =* 0.054), indicating that the positive effects of empowerment education on dairy intake behavior had yet to show up at 1-week post-intervention. However, the experimental group’s score at post-12 weeks (T2) was higher compared to the control group, with the difference being statistically significant (*p* < 0.01), indicating that the positive effects of empowerment education on dairy intake behavior was only significant at 12 weeks post-intervention.

## 4. Discussion

In our study, the concept of empowerment was applied in order to develop strategies for educating older women on daily dairy intake. According to the research results, the experimental group’s daily dairy intake self-efficacy, intention, and behavior improved significantly from T0 to T1, and from T0 to T2, indicating that the empowerment education program had effectively improved the dairy intake self-efficacy, dairy intake intention, and daily dairy intake behavior of the older women. These findings are consistent with those reported by Hsiao et al. (2016) [29], who developed empowerment education intervention measures for kidney transplant patients and found that these measures effectively improved the self-care behavior of such patients.

A six-week empowerment education program was implemented in our study. Partnerships characterized by equal trust were first established since they were the most important component of the program. With these partnerships, the participants became willing to share their thoughts with the researchers, who then utilized open communication to awaken the participants’ intrinsic motivations and provided them with opportunities to reflect on the health effects of dairy intake. Having become empowered to make changes, the participants became willing to make change-related decisions. In 1997, Bandura developed the social learning theory, which proposes that the strength of individual self-efficacy can be influenced through means of performance accomplishments, vicarious experiences, verbal persuasion, and emotional arousal; one of these means, verbal persuasion, is defined as the positive verbal encouragement that an important individual offers to another individual, with the aim of cultivating the latter’s belief that he or she is capable of executing a particular behavior [34]. During the empowerment education classes, the researchers utilized verbal persuasion by continually offering encouragement and praises, such that the participants started to feel that they were capable of acquiring the relevant information. When the participants received encouraging words, they became more assured that they were making progress and applying their skills, which, in turn, strengthened their self-efficacy and intrinsic motivation. Emotional arousal and self-efficacy are negatively correlated; when an individual experiences stress and anxiety, he or she is more likely to respond emotionally or physiologically, which causes the individual to doubt his or her abilities and reduces his or her confidence in executing new behaviors. However, an individual’s self-efficacy can be strengthened by providing outside care and support [34]. In our study, listening and care were utilized to create a supportive environment in which patients felt supported. Telephone counseling can encourage them to continue to change their behavior and answered their question. Through the group discussions that were conducted, the participants came to realize that they shared common experiences and subsequently provided each other with encouragement and support. The psychological support that the participants received alleviated their feelings of anxiety and unease, while also helping to strengthen their dairy intake self-efficacy. Performance accomplishments refer to the positive or negative feedback that an individual receives after completing a certain action, and this will determine whether his or her self-efficacy strengthens or weakens [34]. In one study involving 199 community-dwelling older adults, Park et al. (2017) found that self-efficacy helped to strengthen the calcium supplementation and vitamin D intake behaviors of these older adults, and that successful experiences were effective in raising self-efficacy [35].

Zou’s (2019) study on older Chinese adults with hypertension pointed out that in addition to the family and personal factors, community health education workshops, and friends were important factors that promote healthy eating [36]. Our study transferred valuable knowledge about the consumption of dairy products through an empowerment program and used cooking activities to allow participants to make dairy products together and share recipes, so that they learned many strategies to make changes in their diet, which can effectively promote the intention to consume dairy products. Moreover, the researchers assisted the participants in successfully resolving their dairy intake problems and sharing their feelings and experiences during the group discussions. This approach also allowed the participants to learn problem-solving skills, particularly the older women who were only educated to elementary level, which limited their ability to acquire knowledge through media or printed sources. In the study, these older women were able to learn through the sharing of experiences, which significantly raised their confidence and, consequently, their self-efficacy. These findings are consistent with those from the study conducted by Park et al. (2017) [35], who found that self-efficacy can facilitate the execution of calcium supplementation and vitamin D intake behaviors by community-dwelling older adults; a study carried out by Qi et al. (2011), who conducted a self-efficacy program for 83 older Chinese adults found that their osteoporosis prevention behaviors were positively correlated to their exercise self-efficacy [37].

Furthermore, after completing the intervention, the experimental group’s daily dairy intake self-efficacy and intention continued to increase over 12 weeks. This could be attributed to the success of the study’s empowerment education program in raising the awareness of the older women participants regarding the importance of dairy intake and facilitating their self-reflection on obstacles to dairy intake. Consequently, the participants developed the intrinsic motivation to independently make changes to their behaviors, which helped them to maintain and gradually increase their daily dairy intake self-efficacy and intention over time. This result was similar to the findings of Zou (2019), which pointed out that intrinsic motivation was an important facilitator of improving hypertension and diet [36]. With respect to daily dairy intake behavior, significant improvements in the experimental group were not observed at 1-week post-intervention but showed up at 12 weeks post-intervention. Behavioral change is a gradual process that requires one’s behavioral intentions to be strengthened first. Ajzen’s (1985) theory of planned behavior considers healthy behavior from the perspective of an individual’s behavioral intentions and proposes that an individual would only execute a behavior after he or she has developed strong behavioral intentions [38], that is, a behavioral intention is required in order for an individual to execute a certain behavior. For this reason, it is crucial that we raise awareness among older women regarding the necessity of daily dairy intake. For older women who have yet to experience any symptoms of osteoporosis, the intention to make behavioral intention changes with regard to their daily dairy intake behavior—which is essential to preventing osteoporosis—will only develop when they gain a clear understanding of osteoporosis and its potential effects. Therefore, an individual’s behavioral intention must be strengthened in order for him or her to execute a behavior, and in this respect, our study has achieved significant outcomes in relation to the daily dairy intake intentions of older women, who experienced an increase in their daily dairy intake intentions over time. Although no significant improvement to the older women’s dairy intake behavior was identified at 1 week post-intervention, it was later observed at 12 weeks post-intervention, indicating that behavioral changes require time and only occur after an individual’s behavioral intentions have been strengthened. Dairy products are a possible food to maintain bone health, because it is an important source of calcium and protein, which can reduce bone loss and reduce the incidence of fractures [18,19]. Evidence suggests that consuming a glass of milk per day in older adults was associated with a lower risk of hip fractures [22]. Our study showed that an empowerment education program could be an effective strategy for increasing dairy product intake behavior among older women. Although this study did not measure bone density, our study participants have significantly improved their dairy intake behavior after the intervention, which helps to improve the bone health of older women.

Our study faced several unavoidable research limitations. First, the participants were all recruited from northern Taiwan, which could affect the generalizability of the research results. Thus, for future studies, participants could be sampled from a variety of locations. Secondly, the older women who participated in the study through open recruitment of samples may be those with stronger health motivations; thence there will inevitably be a selection bias. Thirdly, this study was quasi-experimental, it was impossible to control for interference factors that may have affected the inferences of the results. Regarding recommendations for future research, since the outcomes of the empowerment program were only evaluated up to 12 weeks post-intervention, it is recommended that future studies track and verify the long-term effect over a longer period of time. Secondly, our study only evaluated the post-intervention outcomes of an empowerment education program. Since empowerment is a considerably abstract concept, it is recommended that researchers incorporate qualitative research if they intend to confirm whether a group of participants has been genuinely empowered. The participants could be evaluated during the intervention and post-intervention phases, so as to identify changes in regard to how they perceived their own power.

## 5. Conclusions

The empowerment education was effective in improving older Asian women’s dairy intake self-efficacy and intentions and encouraging them to change their dairy intake behavior. Behavioral changes require time and only occur after an individual’s behavioral intentions have been strengthened. Our findings demonstrate that this program was an effective educational intervention strategy. This study can serve as a reference for community nurses for encouraging dairy intake among older Asian women.

## Figures and Tables

**Table 1:** Empowerment education program.

Lesson	Empowerment Principle	Strategy	Education Program Content
1st week	Awareness awakening Provision of necessary information Open communication Mutual participation	Dialogue and reflection Provision of information Sharing of life experiences	Keeping osteoporosis away Introduction. Lecture: Symptoms and disease process of osteoporosis, and prevention strategies. Discuss individual modifiable risk factors. Sharing sessions.
2nd week	Awareness awakening Provision of necessary information Open communication Mutual participation	Dialogue and reflection Provision of information Sharing of life experiences	Drink more milk to stay healthy Introduction. Lecture: Calcium containing foods, and benefits of calcium foods, effects on bone. Discuss dairy product intake. Sharing sessions.
3rd week	Awareness awakening Open communication Mutual participation	Group motivation Dialogue and reflection Provision of counseling Sharing of life experiences	Cheering for your friends, and yourself Introduction. Participants share life experiences. Jointly develop dairy product intake plans and goals. The group listens and provides advice. Sharing sessions.
4th week	Open communication Mutual participation	Group motivation Dialogue and reflection Provision of counseling Sharing of life experiences	I’m a detective Introduction. Situation drama: detective role simulation, allowing participants to find out dietary problems and conduct group discussions. Discuss dairy product intake plans and provides advice. Sharing sessions.
5th week	Open communication Mutual participation	Group motivation Dialogue and reflection Provision of counseling Sharing of life experiences	My dairy products, my decision Introduction. Cooking activity: participants make dairy products together and share recipes. Discuss dairy product intake plans and provide advice. Sharing sessions.
6th week	Open communication Mutual participation	Group motivation Dialogue and reflection Provision of counseling Sharing of life experiences	Carrying on with my efforts Introduction. Personal sharing of dairy product intake plans and goals. The group listens and provides advice. Sharing sessions.

**Table 2:** Summary of personal characteristics by group in the baseline (*n* = 68).

Variables	Experimental (*n* = 34)		Control (*n* = 34)		*X*^2^/*tp*	
	*n* (%) Mean (SD)		*n* (%) Mean (SD)			
**Age**		74.3 (9.8)		76.9 (2.1)	−0.88	0.37
**Marital status**					8.63	0.07
Single	5 (14.7)		1 (2.9)			
Married	25 (73.5)		28 (82.4)			
Divorced or widowed	4 (1.2)		5 (14.7)			
**Education level**					4.75	0.57
Literate	4 (11.8)		2 (5.9)			
Elementary school	14 (41.2)		13 (38.2)			
Junior high school	2 (5.9)		6 (17.6)			
Senior high school	9 (26.5)		10 (29.4)			
Junior college or higher	5 (14.6)		3 (8.8)			
**Residential status**					6.25	0.10
Living with children	14 (41.2)		17 (50)			
Living with spouse	6 (17.6)		11 (32.4)			
Living alone	14 (41.2)		6 (17.6)			
**Financial condition**					2.38	0.49
Poor	3 (8.8)		3 (8.8)			
Average	15 (44.1)		21 (61.8)			
Sufficiently well off	16 (47.1)		10 (29.4)			
**Self-perceived health**					3.11	0.53
Unhealthy	6 (17.6)		8 (23.5)			
Average	16 (47.1)		11 (32.4)			
Healthy	12 (35.3)		15 (44.1)			
**ESS**		16.5 (5.7)		15.8 (5.8)	0.52	0.59
**MNA**		24.3 (1.6)		23.4 (2.2)	1.96	0.06
**Self-efficacy**		8.8 (3.3)		9.2 (3.9)	−0.50	0.61
**Intention**		10.6 (3.6)		10.4 (3.4)	0.20	0.83
**Behavior** (day/month)		6.8 (9.6)		4.4 (8.1)	1.09	0.27

ESS: Emotional social support; MNA: Mini Nutritional Assessment.

**Table 3:** The two groups’ outcome variables at pretest, post-1 week, and post-12 weeks (*n* = 68).

Variables	Dairy Intake Self-Efficacy			Dairy Intake Intention			Dairy Intake Behavior		
	Beta	SE	*p*	Beta	SE	*p*	Beta	SE	*p*
Experimental									
T1 vs. T0	2.79	0.60	<0.001	1.27	0.43	0.004	2.47	1.07	0.021
T2 vs. T0	3.53	0.66	<0.001	1.59	0.50	0.002	4.29	1.33	<0.001
Control									
T1 vs. T0	0.09	0.08	0.248	−0.38	0.42	0.358	0.32	0.32	0.306
T2 vs. T0	0.06	0.08	0.476	−0.32	0.50	0.514	0.18	0.32	0.579

T0: pretest; T1: post-1 week; T2: post-12 week; SE: standard error; the reference group is the pretest (T0).

**Table 4:** Intergroup differences in outcome variables (*n* = 68).

Variables	Dairy Intake Self-Efficacy			Dairy Intake Intention			Dairy Intake Behavior		
	Beta	SE	*p*	Beta	SE	*p*	Beta	SE	*p*
Group E vs. C	−0.44	0.86	0.60	0.17	0.84	0.83	2.35	2.12	0.26
Time									
T1 vs. T0	0.08	0.07	0.24	−0.38	0.41	0.35	0.32	0.31	0.30
T2 vs. T0	0.05	0.08	0.47	−0.32	0.49	0.51	0.17	0.31	0.57
Group * Time									
Group * (T1 vs. T0)	2.70	0.60	<0.001	1.64	0.60	<0.01	2.14	1.11	0.054
Group * (T2 vs. T0)	3.47	0.66	<0.001	1.91	0.70	<0.01	4.11	1.36	<0.01

E: experimental group; C: control group; T0: pretest; T1: post-1 week; T2: post-12 week; SE: standard error; the reference group is the control group and pretest (T0); Group * Time: Interaction effect, the meaning of the interaction is {(experimental group post-test—pretest)—(control group post-test—pretest)} which is the comparison of the improvement range between the two groups.

## Data Availability

The data presented in this study are available on request from the corresponding author.

## Supplementary Materials

The following are available online at https://www.mdpi.com/article/10.3390/ijerph18094659/s1, Table S1. Empowerment education program.
