# Supplementary material for: The Effects of Empowerment Education on Daily Dairy Intake in Community-Dwelling of Older Asian Women
Source: Int J Environ Res Public Health. 2021 Apr 27;18(9):4659. doi: 10.3390/ijerph18094659 (PMC8124786; doi:10.3390/ijerph18094659)
Supplement: Supplementary file 1 [file ijerph-18-04659-s001.zip › ijerph-1170103-supplementary.pdf]

Supplementary File

Table S1. Empowerment education program.

Lesson	Education program	Content
1st week	**Keeping osteoporosis away**<br>1. Introduction.<br>2. Lecture：Symptoms & disease process of osteoporosis, and prevention strategies.<br>3. Discuss individual modifiable risk factors<br>4. Sharing sessions.	1. The first section (40mins)<br>The participants and the educator will have a dialogue before the activity begins, this may eliminate the structure of the leader- member relation. Then they will introduce their selves to each other, to build up the relationship.<br>2. The second section (30mins)<br>We invited a nutrition professional to give a keynote speech of "symptoms & disease process of osteoporosis, and prevention strategies". We hope this can provide the older adults correct information on osteoporosis and information of daily life, to increase the health education and healthy life index.<br>3. The third section (50mins)<br>After the speech, participants share the difficulties and experiences about osteoporosis in daily life and will carry on the dialogue and reflection.
2nd week	**Drink more milk to stay healthy**<br>1. Introduction.<br>2. Lecture：Calcium containing foods, and benefits of calcium foods, effects on bone.<br>3. Discuss dairy product intake.<br>4. Sharing sessions.	1. The first section (40mins)<br>The participants and the educator will have a dialogue before the activity begins. The educator will ask participants, "How about the past week?" and the participants were invited to take and share. Enhancing participants' conscious awareness and evolving a partnership through dialogue.<br>2. The second section (30mins)<br>We invited a nutrition professional to give a keynote speech on "calcium-containing foods, and benefits of calcium foods, effects on bone".<br>3. The third section (50mins)<br>After the speech, the speaker discussed with the participants the status of dairy product intake and show different kinds of milk and dairy products to let the participants understand and taste them, and will carry on the dialogue and reflection.
3rd week	**Cheering for your friends, and yourself**<br>1. Introduction.<br>2. Participants share life experiences.<br>3. Jointly develop dairy product intake plans and goals.<br>4. The group listens and provides advice.<br>5. Sharing sessions.	1. The first section (30mins)<br>The participants and the educator will have a dialogue before the activity begins. The educator will ask participants, "How about the past week?" and the participants were invited to take and share. The aim was to continue to enhance participants' conscious awareness and strengthen partnership through dialogue.<br>2. The second section (60mins)<br>Everyone shared their experiences about drinking milk, including the barriers of drinking milk and methods to overcome them.<br>3. The third section (30mins)<br>The educator discussed with the participants the plans and goals of dairy product intake. Everyone listens and provides advice, then they will carry on the dialogue and reflection.
4th week	**I'm detective**<br>1. Introduction.<br>2. Situation drama: detective role simulation, allowing participants to find out dietary problems and conduct group discussions.<br>3. Discuss dairy product intake plans and provides advice.<br>4. Sharing sessions.	1. The first section (30mins)<br>Before the activity begins, the participants were invited to take by educator, then asked participants, "How's it going the past week?" and participants talked about their feeling about dairy product intake. The educator must maintain empathy, understanding, and trust towards the participants, offering myself as a resource for listening and mental support.<br>2. The second section (60mins)<br>First, will introduce the activity content about "I am Detective". In this activity, each participant simulates the role of a detective. The leaders and volunteers perform a live scene. At the end of each scene, they use the insight of the elders to that find out the problems of the diet.<br>3. The third section (30mins)<br>The educator discussed with the participants the plans and goals of dairy product intake. We use group discussion to discuss dairy product intake plans and provides advice.

5th week	**My dairy products, my decision**<br>1. Introduction.<br>2. Cooking activity: participants make dairy products together and share recipes.<br>3. Discuss dairy product intake plans and provides advice.<br>4. Sharing sessions.	1. The first section (30mins)<br>Before the activity begins, the participants were invited to take by educator, then asked participants, "How's it going the past week?" and participants talked about their feeling about dairy product intake. The educator still maintained empathy and understanding for participants and provided listening and mental support.<br>2. The second section (60mins)<br>First introduce the activity of "My dairy products, my decision", then participants will be divided into groups and cook together, they will make some diary food like yogurt and cheese, exchange the cooking skills and experience, that the participants know there are many recipes about diary food.<br>3. The third section (30mins)<br>Share the thought of the cooking activity. Then the educator discussed their dairy product intake plan with the participants and provided suggestions through group discussions.
6th week	**Carrying on with my efforts**<br>1. Introduction.<br>2. Personal sharing of dairy product intake plans and goals.<br>3. The group listens and provides advice.<br>4. Sharing sessions.	1. The first section (60mins)<br>Before the activity begins, the educator asked participants, "How's it going the past week?", then the participants were invited to share their feelings about participating in the course over the past five weeks.<br>2. The second section (60mins)<br>Participants share their own plans about the intake of dairy products and goals over the past three weeks with everyone. The group listens and provides advice. At last, they will build up a social support network about dairy products to share recipes and life experiences with each other.
